# Current remote sensing approaches to monitoring forest degradation in support of countries measurement, reporting and verification (MRV) systems for REDD+

**DOI:** 10.1186/s13021-017-0078-9

**Published:** 2017-04-17

**Authors:** Anthea L. Mitchell, Ake Rosenqvist, Brice Mora

**Affiliations:** 1 School of Biological, Earth and Environmental Sciences, The University of New South Wales, Kensington, NSW 2052 Australia; 2 Solo Earth Observation, LLC, Tokyo, Japan; 3 GOFC-GOLD Land Cover Office, Wageningen University, Wageningen, The Netherlands

**Keywords:** Degradation, Disturbance, Measurement reporting and verification, REDD+, Carbon emissions, Monitoring, Time-series, Forests, Above-ground biomass

## Abstract

Forest degradation is a global phenomenon and while being an important indicator and precursor to further forest loss, carbon emissions due to degradation should also be accounted for in national reporting within the frame of UN REDD+. At regional to country scales, methods have been progressively developed to detect and map forest degradation, with these based on multi-resolution optical, synthetic aperture radar (SAR) and/or LiDAR data. However, there is no one single method that can be applied to monitor forest degradation, largely due to the specific nature of the degradation type or process and the timeframe over which it is observed. The review assesses two main approaches to monitoring forest degradation: first, where detection is indicated by a change in canopy cover or proxies, and second, the quantification of loss (or gain) in above ground biomass (AGB). The discussion only considers degradation that has a visible impact on the forest canopy and is thus detectable by remote sensing. The first approach encompasses methods that characterise the type of degradation and track disturbance, detect gaps in, and fragmentation of, the forest canopy, and proxies that provide evidence of forestry activity. Progress in these topics has seen the extension of methods to higher resolution (both spatial and temporal) data to better capture the disturbance signal, distinguish degraded and intact forest, and monitor regrowth. Improvements in the reliability of mapping methods are anticipated by SAR-optical data fusion and use of very high resolution data. The second approach exploits EO sensors with known sensitivity to forest structure and biomass and discusses monitoring efforts using repeat LiDAR and SAR data. There has been progress in the capacity to discriminate forest age and growth stage using data fusion methods and LiDAR height metrics. Interferometric SAR and LiDAR have found new application in linking forest structure change to degradation in tropical forests. Estimates of AGB change have been demonstrated at national level using SAR and LiDAR-assisted approaches. Future improvements are anticipated with the availability of next generation LiDAR sensors. Improved access to relevant satellite data and best available methods are key to operational forest degradation monitoring. Countries will need to prioritise their monitoring efforts depending on the significance of the degradation, balanced against available resources. A better understanding of the drivers and impacts of degradation will help guide monitoring and restoration efforts. Ultimately we want to restore ecosystem service and function in degraded forests before the change is irreversible.

## Background

Forest degradation, together with deforestation, are placed second to burning of fossil fuels in terms of contributing to greenhouse gas (GHG) emissions [81]; a key driver of global climate change [44]. Deforestation, forest degradation and peat land fires accounted for around 15% of global anthropogenic emissions of carbon dioxide (CO_2_) between 1997 and 2006 [91]. The drivers and intensity of degradation vary by region [48], but the impact of forest loss and degradation can be felt at all scales, from global climate change to declining economic value of forest resources and biodiversity and threatened local livelihoods. Urgent and decisive action to curb the extent of deforestation and forest degradation, and promote the enhancement of carbon stocks through regeneration and afforestation, and thus better accounting of CO_2_ sources and sinks is paramount.

To address this issue, the United Nations Framework Convention on Climate Change (UNFCCC) has adopted a mechanism for reducing emissions from deforestation and forest degradation and the role of conservation, sustainable management of forests and enhancement of forest carbon stocks in developing countries (REDD+), which would provide financial incentives for emissions reductions [68]. In order to implement REDD+, countries are required to establish national measurement, reporting and verification (MRV) systems within an existing or newly established National Forest Monitoring System (NFMS) that provide annual, national estimates of changes in forest carbon stocks and emissions and that are reported biennially [41, 43]. The recommendation of the Intergovernmental Panel on Climate Change (IPCC) is to use a combination of Earth Observation (EO) data and field-based inventory to estimate the forest area, carbon stocks and changes [25, 41]. The MRV system should provide estimates that adhere to IPCC principles of transparency, comparability, consistency, completeness and accuracy [41], with emissions estimated from all relevant activities [22]. A framework definition of forest degradation is adopted once thresholds for time, minimum carbon loss and possibly minimum area are determined [42]. While there is no set criteria, methods should be adopted that achieve unbiased estimates with the lowest uncertainty as is practicable [42].

Presently, parts of an MRV system (i.e., deforestation) can be operated using available satellite and forest inventory data. However, data on quantitative changes associated with forest degradation are generally missing, and in many developing countries there is low capacity for monitoring of, and reporting on emissions from degradation (and removals from regrowth and afforestion) on a national level [31]. Traditional field-based National Forest Inventories (NFI) allow for estimates of change in growing stock and biomass, and do so primarily by periodic field measurement using permanent sample plots (PSPs; [17]). Not all NFIs are initially designed for carbon stock assessments however, and measurements may not extend to all significant carbon pools [31]. Sampling specifications are ideally defined on the basis of the required precision, however, more often than not, are governed by time constraints and labour costs. There are difficulties of access in some areas and it may be more cost-effective to reduce sampling intensity in these areas and concentrate sampling effort on a few select classes. The use of terrestrial laser scanners (TLS) and drones may speed up the process of collecting data from which structural attributes can be estimated. Access to country specific models to estimate forest carbon stocks also presents a significant challenge. The allometric equations used to estimate tree volume and biomass are not available for all tropical forest types and species [17], and additional measurements by destructive harvesting would increase the survey costs [8]. Arguably the greatest challenge faced by countries is the lack of Government endorsed programs that instil a dedicated effort to consistent monitoring of forests at national scale [17]. Maintaining institutional capacity and drive is key to assessing the state of the forest resource with a view to sustainable management. Field inventory should ideally be multi-purpose and collect data to suit a range of stakeholders and so maximise use and investment.

Complementing field-based inventories with EO data allows for greater areal coverage and reduces the burden on field survey. EO data can be acquired wall-to-wall or on a sampling basis (in particular for very high resolution, VHR, data) across the region/nation of interest. Satellite observations can be used to estimate the area of forest classes (including degraded and intact forest states), for which volume and biomass densities can be extrapolated using field-based measurements [17]. Repeat observations of both EO and field data allow for ongoing assessments of changes in forest carbon stocks. Estimates of forest structure and above ground biomass (AGB) are also possible using SAR and LiDAR data. Appropriate satellite EO data that spans several decades is also available at moderate resolution from both optical, and over a shorter time period, SAR sensors. These data allow for longer term assessment of forest dynamics in response to both anthropogenic and natural disturbances. An integrated approach that combines multi-sensor EO and in situ data could form part of a systematic framework for monitoring changes in forest cover and carbon stocks. This would allow the implementation of a more complete MRV system, whereby the disturbance history, i.e., degradation type and long-term loss of carbon stocks in forest land, is needed to account for emissions arising from forest degradation. Offsetting these losses with accounting of long-term carbon gain incurred through afforestation and sustainable management practices may be an important consideration.

The task of mapping forest degradation is far more challenging than for deforestation [33]. Forest degradation (as well as enhancements of carbon stocks) is typically manifested through a change in forest structure, often subtle, and carbon losses (and gains) are smaller and more difficult to detect and quantify than deforestation using remote sensing where often significant reductions in canopy cover are observed. There are some degradation processes that defy detection by remote sensing altogether, including, for example, fuel wood extraction and understorey grazing [82]. While there is a loss of AGB associated with these activities, the forest canopy remains untouched. Estimates of AGB loss in these cases are best collected by forest inventory or production/consumption surveys [17].

Current remote sensing monitoring approaches can be divided into two main categories: (1) the detection of degradation (or proxies) which could form part of an early warning system [e.g., 49, 72, 74], and (2) quantification of loss (or gain) in AGB [e.g., 63, 65, 84], which countries need to include in their emissions reporting. The EO data requirements can be expected to vary depending on the type of degradation or proxy to be monitored. Guidance is gradually being formulated on what observations are required, the timeframe over which to monitor and how best to extract the information [e.g., 22, 25]. Research on forest degradation mapping methods is considered a high priority [21]; it is a crucial missing link in countries’ carbon accounting systems. The need for information on forest degradation goes beyond that of REDD+, with countries wanting effective strategies to monitor the state of their forest resources and better inform management decisions and restoration activities, track illegal logging activities, and protect biodiversity and local livelihoods [9, 17, 64].

There is a lack of knowledge and awareness of EO technology and capability to assess AGB and canopy level change associated with degradation. The purpose of this paper is to review current remote sensing approaches to forest degradation monitoring in the context of MRV and REDD+. The operational readiness of current approaches and EO technologies is evaluated. The paper concludes by identifying important gaps and research and development (R&D) needs to advance methods to operational status for use by countries in their national forest monitoring systems.

### Forest degradation defined

Forest degradation can be defined in innumerable ways, and indeed one man’s concept of forest management may be another’s source of degradation. In the context of the UNFCCC REDD+, forest degradation entails any direct, anthropogenic-induced and persistent loss in carbon density over time in forest land remaining forest [42]. Degradation should be considered in continuum within the bounds of ‘forest’ definitions based on, for example, height and canopy cover and will never reach land use change [56]. A key challenge lies in first defining the baseline carbon stock, against which change (i.e., persistent decline) can be monitored [26].

The impact of degradation varies from fine-scale structural changes in canopy cover and height [19, 34], or subtle disruptions to ecosystem services, to broad-scale loss of biomass [14, 64]. These changes can occur over a range of spatial and temporal scales. Degraded forest may assume a similar canopy cover to intact forest, but have lower biomass, in some cases reduced by up to 75% [89]. Different types of forests will respond differently to change, with variable recovery rate, depending on the location and type, intensity and extent of degradation. As such, a single monitoring strategy may not be appropriate for broad-scale application; rather a customised region-specific approach is required.

### Forest degradation: the global picture

The main driver of degradation in sub/tropical countries is unsustainable logging [38]. Rapid economic and population growth, expansion of commercial agriculture and complacency in sustainable forestry practices are key contributing factors. The commercial demand for timber and unsustainable logging practices has introduced a cycle of degradation with persistent loss of biomass and canopy cover across insular South East Asia and Latin America [48, 64]. Shifting cultivation, over-grazing, fire, fuel wood collection and charcoal production have also resulted in degradation in large parts of Africa [38].

**Fig. 1:**
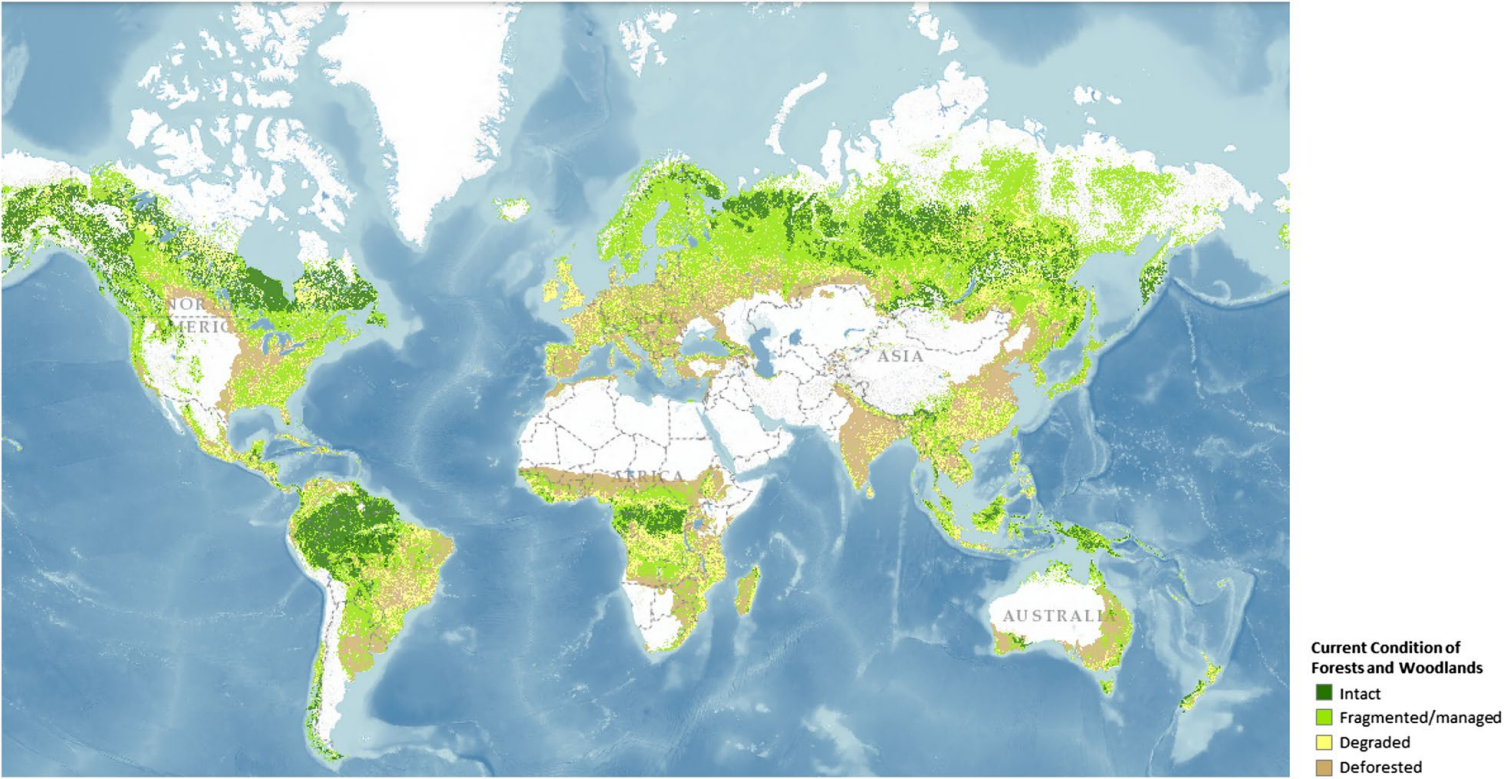
The global forest condition [68], as visualised using satellite derived and modelled current and potential forest cover [88]

The Atlas of Forest Landscape Restoration Opportunities [88] is a world-first attempt at characterising the spatial extent of degraded forests worldwide and areas of restoration potential. Forest condition (Fig. 1; [70]) was mapped at 1 km resolution by comparison of current (largely MODIS derived) and potential (modelled) forest cover change estimates. Forest condition and land use data were used to identify opportunities for restoration on degraded lands. The derived maps provide a global overview and may assist in identifying areas for more detailed analysis.

Recently released global products have quantified forest extent and change at unprecedented scale, and provide valuable data for understanding forest trends and the implications of disturbance on carbon stocks, biodiversity and human livelihoods [24]. Global tree cover extent, loss and gain between 2000 and 2012 was mapped using Landsat derived time-series metrics [27]. The greatest total forest loss and gain occurred in the tropics where deforestation was dominant, with an estimated 32% of global forest loss occurring in tropical rainforest and mostly in South America. High rates of forest loss were also experienced in Eurasia and Africa. The datasets provide a long-term consistent record of change, from which degradation, drivers, albeit indirectly, and policy actions can be determined.

## Main text

### Approaches to monitoring forest degradation

Long-term and consistent monitoring is key to discriminating degraded and intact forest, and separating change due to anthropogenic impacts and seasonal/cyclic change [64]. The type of degradation may under certain circumstances be identified on a single-date image [e.g., 86], however, a time-series of satellite data (e.g., monthly or intra-annual observations) are generally needed to better capture the dynamics in forest cover and carbon stock changes. This review considers two key approaches: (1) the detection and characterisation of degradation as indicated by changes in canopy cover, or proxies which could form part of an early warning system, and (2) quantification of loss (or gain) in AGB, as an intermediate step to reporting on emissions. The various approaches are summarised at the end of the Section in Table 1.

**Table 1:** Summary of approaches to forest degradation monitoring

	Method										LiDAR		Optical								SAR						
Mapping approach	Dense time-series tracking	Change detection	Vegetation indices	Data transforms	Spectral Mixture Analysis	Classification	Interferometry	Modelling	Data fusion	Visual interpretation	ICESat GLAS	Airborne LiDAR	MODIS	CBERS	Landsat	SPOT	Sentinel-2	RapidEye	Quickbird	IKONOS	ALOS-1/2 PALSAr-1/2	ENVISAT ASAR	SRTM	TerraSAR-X	TanDEM-X	Cosmo-SkyMed	GeoSAR
(1) Detection and characterisation of degradation type																											
Forest disturbance mapping	●		●												●												
	●		●												●												
	●	●				●									●												
	●		●												●												
		●		●											●												
					●					●				●	●												
					●								●														
					●													●									
					●	●									●												
	●		●										●														
			●			●										●											
		●																			●						
						●									●						●			●			
Identification of canopy gaps					●														●								
			●												●		●										
		●																						●			
	●	●																				●		●			
						●															●	●		●			
						●																		●			
						●						●															
								●		●		●															
Proxies						●				●					●					●							
										●														●			
										●																	●
						●															●			●			
										●		●															
(2) Quantification of carbon stock changes																											
Tracking of secondary and degraded forest dynamics	●					●									●												
						●									●						●						
						●		●				●															
Canopy height change		●						●				●															
								●			●																
							●														●						
							●																●			●	
							●																●		●		
							●																●		●		
AGB change								●				●															
								●				●															
								●				●															
								●				●															
								●	●		●																
								●	●		●																
								●													●						
							●	●															●		●		
							●	●															●		●		

	Study specifics				
Mapping approach	Scale^a^	Country	Map resolution (m)	Map accuracy/RMSE	References
(1) Detection and characterisation of degradation type					
Forest disturbance mapping	R	Ethiopia	30		[15]
	R	USA	30		[62]
	R	USA	30	98% (prod acc.), 86% (user acc. spatial), 80% (user acc. temporal), 90% (overall acc. LC)	[96]
	N	Australia	30		[54]
	N	USA, Canada	30	44.6 ± 5.8% (omission), 27 ± 4.5% (commission)	[61]
	N	Brazil	20–30		[90]
	R	Brazil	250		[80]
	R	Indonesia	6.5	91.5%, 0.87 (Kappa)	[19]
	R	Central Africa	30	87%	[34]
	R	USA	250	88%	[87]
	R	Tanzania	10–20	20–25% misclassification (20% cover class)	[37]
	R	Brazil	25 m		[35]
	R	Indonesia	<30	79.6%	[94]
Identification of canopy gaps	R	Gabon, Congo	2.4		[72]
	R	Cambodia	10, 30		[51]
	R	Panama	1		[50]
	R	Sumatra, Brazil	1–3	93.4% (FAR 2.3% at 95% conf int.)	[36]
	R	Congo	<10	53.6%, 100% (user acc.)	[74]
	R	Brazil	2	86%	[7]
	R	Peru			[5]
	R	Brazil	5		[2]
Proxies	R	Congo			[52]
	R	Norway	10		[83]
	R	PNG	5		[95]
	R	Congo		95% (user acc.), 70.4% (overall acc., L-band), 100% (user acc.), 53.6% (overall acc., X-band)	[74]
	R	Amazon			[2]
(2) Quantification of carbon stock changes					
Tracking of secondary and degraded forest dynamics	R	Brazil	30	88% (Kappa 0.62)	[29]
	R	Australia	25	77.8% (Kappa 0.69)	[59]
	R	Costa Rica	20	RMSE RH100 1.34 m (r^2^ = 0.69, p < 0.001) Corr. with ISODATA1 and early stage (97%), ISODATA3 and late stage (99%), ISODATA2 and intermediate stage (56%) RH100/RH75 r^2^ 0.79 (late), r^2^ 0.73 (intermed.), r_2_ 0.72 (early) RH50 r^2^ 0.3 (intermed.)	[11]
Canopy height change	R	Brazil	1		[2]
	R	Gabon		r^2^ = 0.83, RMSE 3.3 m, n = 95	[65]
	R	Australia			[55]
	R	Cameroon, DRC	2	75–82%	[13]
	R	Tanzania			[84]
	N	Uganda		0.9 mm (bias), 8–16 mm (bias upslope)	[85]
AGB change	R	Kalimantan		r^2^ = 0.77 (PPR 54.2 Mg ha^−1^), r^2^ = 0.81 (PPR 47.4 Mg ha^−1^)	[16]
	R	Brazil		R^2^ = 0.7, SE 41.5 Mg ha^−1^	[2]
	R	Kalimantan		r^2^ = 0.88, RMSE ± 13.8 Mg 0.13 ha^−1^	[46]
	R	Norway	14	95.7–97.8% (classification of deforestation and untouched classes), 56.3–69.2% (degradation classes) AGB: SE 5–8.4 Mg ha^−1^, r^2^ = 0.88–0.98 SE reduced by 18–84% using LiDAR, largest gains in degradation class (73–84%)	[67]
	R	Kalimantan	50/100	SE 53.2 Mg ha^−1^ (n = 51 @50 m), 49.1 Mg ha^−1^ (@100 m)	[71]
	R	Brazil	30	p < 0.0001, R^2^ = 0.6, N = 26	[29]
	R	Mozambique		RMSE 8.7–10.9 MgC ha^−1^ Mean error 9.8 ± 0.7 MgC ha^−^1, mean absolute bias 1.6 ± 0.1 MgC ha^−1^	[78]
	R	Tanzania	10	67.2 Mg ha^−1^ (51%), bias −5.5 Mg ha^−1^ (−4.2%), precision 67 Mg ha^−1^ (51%)	[84]
	N	Uganda		±8.5 Mg ha^−1^ (95% conf int.)	[85]

^a^Scale: ‘N’ National, ‘R’ Regional

### Detection and characterisation of degradation

The various approaches attempt to stratify the forest by degradation type or intensity, or use proxies as an indicator of change. Forest disturbance, arising from logging, burning, disease or insect infestations, can be monitored by remote sensing approaches that detect changes in canopy cover. Current methods analyse changes in spectral response, spectral fractions and indices, and try to separate degraded and intact forests. Selective or high intensity logging leads to fragmentation of the forest canopy, and remote sensing methods are aimed at detecting canopy gaps and clearings. Proxies, in the form of forest roads, trails and log decks provide evidence of clearing activity, and a range of methods, including spectral fractions, spatial filtering and proximity metrics are employed to identify and map their progression. In this section, reference is made to sensors that observe at coarse (>100 m), moderate (10–100 m), high (5–10 m) and Very High spatial Resolution (VHR, <5 m). The spatial resolution of specific satellite sensors is indicated in brackets.

#### Forest disturbance mapping

Multiple, complementary satellite observations can be used to construct a long time-series to track forest disturbance. Following detection of disturbance in the signal, the unit change (e.g., percent) in a vegetation metric, relative to a reference condition, is estimated. One such approach is the pixel-based Break detection For Additive Seasonal Trends (BFAST) Monitor [92], which models the expected behaviour of a time-series and identifies those pixels that deviate significantly as breakpoints. The magnitude of the detected change is related to the type of change. BFAST was applied successfully to a time-series of Landsat Normalized Difference Vegetation Index (NDVI) images in Kafa zone, Ethiopia [15], to identify deforestation (high negative change magnitude breakpoints) and forest degradation (low magnitude breakpoints). Breakpoints could be aggregated to an annual scale and so used to report on forest degradation trends [15]. Irrespective of sensor type, a continuous data record and high quality forest mask to eliminate false positives (e.g., crop phenology) are critical to use.

Change detection using pre- and post-disturbance imagery is generally limited to the detection of broad-scale change. Change detection is more powerful however, when the signal is analysed over a long time period, with improved signal-to-noise ratio and detection of subtle change in forest cover and condition [47]. Built on this concept, LandTrendr (Landsat-based Detection of Trends in Disturbance and Recovery) [47] extracts spectral trajectories of change using annual Landsat data stacks, and applies temporal segmentation and fitting strategies that capture both slow processes (e.g., regrowth) and abrupt change events (e.g., harvesting). The methods are generally applicable at national scale, and allow reconstruction of the disturbance history and continuous forest monitoring with more recent observations [39]. Meigs et al. [62] applied Landtrendr to a time-series of Landsat derived Normalized Burn Ratio (NBR) images to assess forest dynamics in response to insect outbreak in conifer forests in western North America. By observing a wide range of spectral trajectories, a more complete picture of change (i.e., defoliation, mortality and recovery regime) across different forest types is gained [62]. Saturation effects in high productivity forests with an abundant understorey may be limiting, and detectability along productivity gradients should be explored [62].

Zhu and Woodcock [96] developed the Continuous Change Detection and Classification (CCDC) algorithm which also exploits the high temporal frequency of Landsat data to detect land cover change. A change pixel is identified where change has occurred in three consecutive observations. Following the detection of change, the land cover is mapped using a random forests classifier. A trial of CCDC in New England allowed the detection of land cover change with a producer’s accuracy of 98% and user accuracies of 86% (spatial) and 80% (temporal; [12]). An overall accuracy of 90% was observed in the resulting 16-class land cover map. Near-real time change monitoring will be possible by combining Landsat and Sentinel-2 observations [96]. VegMachine is an Australian-based operational system for identifying national trends in forest cover change, including disturbance and recovery in response to wild fire, disease and logging [54]. The method uses a simple woodiness index applied to time-series Landsat data. The timing, direction, magnitude and extent of changes in vegetation cover are mapped in the process. User intervention is required to attribute the changes with a direct cause.

Data transforms aim to reduce and rescale the spectral dataset and, in so doing, maximise the spectral separability between disturbed and undisturbed forest. Masek et al. [61] demonstrated national-scale application of the Disturbance Index [28] by producing a map of forest disturbance for the USA and Canada using decadal change in Landsat DI. Landsat data from two epochs, 1990 (with images acquired between 1986 and 1992) and 2000 (1999–2001) were analysed. In Washington State, it was possible to identify recent clearcuts, logging roads and areas of regrowth forest from past clearing events. The method appeared biased towards underestimating national forest disturbance by 17.6 ± 7.4% (uncertainty at 90% confidence interval; [61]). The lengthy time interval (decadal) in the study was limiting, with around 30–60% of disturbance not mapped. It was suggested that a shorter interval (<2 years) was needed to detect and map subtle disturbance and rapid recovery incurred through, for example, forest thinning or insect defoliation [61].

At the pixel level, degraded forests comprise mixed fractions of vegetation, dead wood, soil and shade (‘mixels’). These fractions can be isolated by Spectral Mixture Analysis (SMA) and subsequently classified to reveal the extent and degree of degradation. INPE’s (National Institute for Space Research Brazil) DEGRAD system is one of few national operational systems for forest degradation monitoring [40]. Logging impacts and progressive forest degradation are identified using multi-date, contrast-enhanced Landsat and CBERS-2 (20 m spatial resolution) imagery and derived soil and vegetation fraction ratios [90]. Using coarser resolution MODIS data (250 m), [80] demonstrated an operational approach to monitoring forest degradation due to fire in Mato Grasso, Brazil. Deforested and burned areas were mapped using the soil and shade fractions respectively. Finer scale change, including selective logging could not be mapped. The cumulative impact of low impact logging and fire in peat swamp forest in central Kalimantan, Indonesia, was observed as an increase in the soil fraction in a time-series of 3 RapidEye images [19], and mapped with an overall accuracy of 91.5% (Kappa 0.87). Degradation due to selective logging was mapped using a minimum distance classifier applied to a time-series of Landsat data acquired over test sites in Cameroon and the Central Africa Republic at overall accuracies of 87% [34]. The characteristic peak in the soil fraction was only visible for 2 years in the time-series over Cameroon. Regrowth can be identified using the peak in the green vegetation fraction, which can maintain higher values than that of intact forest for 2–10 years following the logging event [34]. Timely and high spatial resolution remote sensing observations (in accordance with vegetation recovery rates) and an accurate forest baseline map against which to observe change, were critical inputs to the studies.

A time-series of vegetation indices derived from optical data can be used to estimate change relative to a reference level and generate a map of the area affected by a particular disturbance. Gypsy month defoliation maps were produced from unsupervised classification and thresholding of a 7-year time-series of MODIS-derived maximum NDVI images in the Mid-Appalachian region of the USA [87]. Pixels that displayed a 4% change or greater in NDVI were classified as defoliated. The use of MODIS daily products outperformed the 16-day composites, with a lower omission rate (0.09 vs. 0.56) and higher overall classification accuracy (88 vs. 79%). The approach provides a useful tool for planning aerial surveys and potential development of a nationwide near-real time monitoring system. At a finer scale, a dense time-series of SPOT-4 (20 m) images was used to map different forest cover percentages for intact and degraded forest in Tanzania [37]. Forest classes were more easily separable with canopy cover changes of 40% or more. Most classification errors were observed in the 10–20% canopy cover class, with a misclassification error of around 20–25%. An improvement in separation capability was demonstrated by simulating results based on Sentinel-2 revisit times [37].

Synthetic aperture radar (SAR) has demonstrated reliable detection of degradation resulting from complete or partial removal of tree cover, and in areas where optical image availability is limited by near-permanent cloud cover. Mapping approaches are reliant on 2D classification of SAR backscatter or automated change analysis applied to calibrated time-series. Hoekman [35] identified shifting cultivation on a multi-temporal stack of ALOS PALSAR dual polarisation data (25 m) in Para State, Brazil. SAR-optical data fusion may also improve the discrimination and mapping of degraded forests. Wijaya [94] used a combination of SAR backscatter and polarimetric features derived from ALOS PALSAR (12.5 m) and TerraSAR-X (6 m) data with Landsat reflectance to identify degraded peat swamp and other forest types in Indonesia. Mapping results were improved in a combined SAR-optical classification, with an overall accuracy of 79.6% compared to using SAR data alone (48.3%).

#### Identification of canopy gaps and clearings

Unsustainable forestry practices, such as selective logging on an all too frequent basis, or high intensity logging, can induce a cycle of long-term reduction in canopy cover and biomass, ultimately rendering the forest degraded [66]. Timber extraction results in often sizeable gaps in the canopy due to felled logs, clearings, roads and log decks. Mapping approaches are reliant on the capacity to detect gaps in, or fragmentation of, the forest canopy to identify degradation activity [4]. Direct observation of canopy damage, small clearings and other structural changes is possible using VHR to moderate (Landsat-like) resolution optical and SAR [32], and also LiDAR data [5]. In optical data, the spectral signature of canopy gaps and clearings is also different to that of surrounding intact forest. This difference can be exploited in SMA (described in the previous section) and maps produced through the classification of fraction images. Rahm et al. [72] were able to map 5 levels of degradation using the percentage difference of bare soil fraction in time-series Quickbird images (2.4 m; 2010 and 2012) in Gabon and Democratic Republic of Congo. Frequent mapping of canopy gaps and clearings is required as the spectral signature changes quite rapidly as the forest regrows, and is typically indiscernible from intact (undisturbed) forest in a matter of <2 years [32]. The means to capture these short-lived disturbance signals in tropical forest will improve with the Sentinel-2 constellation [51]. Langner et al. [51] compared an NBR differential method applied to Landsat-8 and Sentinel-2 data for detecting forest canopy disturbance due to selective logging in central Cambodia. Visual comparison revealed a similar pattern of disturbance in both datasets, however, largely due to purer pixels, the level of detail was greater in the Sentinel-2.

**Fig. 2:**
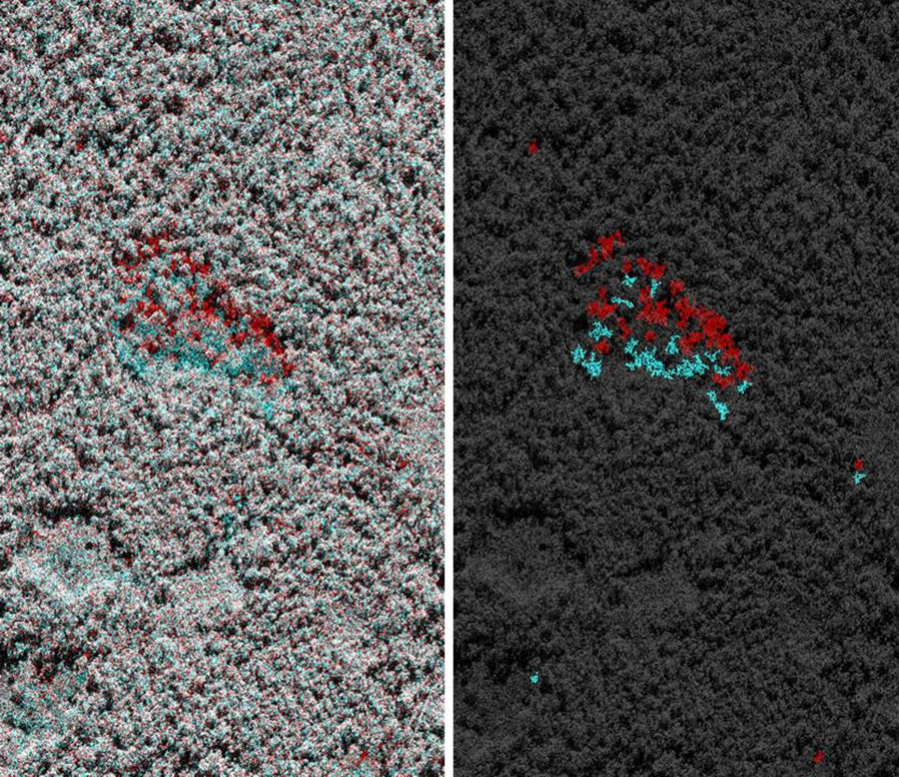
TerraSAR-X Spotlight imagery (Oct 2013 in* red*, Jan 2014 in cyan) and automated change mapping result for Calha Norte, Brazil, showing removal of individual trees through detection of disappearing tree crowns (*red*) and radar shadows (*cyan*); Courtesy of [36]

Time-series approaches applied to calibrated VHR SAR data have been successful in detecting the removal of individual trees [36, 49, 50]. Automated mapping of selective logging activity was demonstrated using TerraSAR-X Spotlight images (1 m) in Panama [50]. Frequent observations by TerraSAR-X (11 day revisit time) allow for independent monitoring of forestry activities, including logging (selective and illegal logging), fire and regrowth [50], the impacts of which may be interpreted as degradation. Near-real time monitoring capability has been realised in several locations in Brazil, Suriname, Guyana and Indonesia, to help tackle illegal logging and encroachment [36]. The loss of individual trees was mapped through the detection of disappearing tree crowns and radar shadows in TerraSAR-X Spotlight data (2 m; Fig. 2). The overall accuracy for a fully automated X-band monitoring system in the Harapan rainforest, Sumatra, was 93.4%, with a false alarm rate (FAR) of 2.3% (i.e., the probability of incorrectly identifying a canopy gap, at 95% confidence level; [36]). Detection error is reduced through the implementation of automated spatio-temporal filtering to minimise speckle and precipitation effects. Hoekman [36] are also investigating wall-to-wall degradation and deforestation monitoring capability using Sentinel-1 data.

VHR data is typically required to detect fine-scale degradation that involves the removal of individual trees. A SAR-based example from the Republic of Congo demonstrates the problem. Here, the area of selectively logged forest was under-estimated by 37.5% in ALOS PALSAR data [74], and poor detection accuracy was observed when using ENVISAT ASAR compared to TerraSAR-X data (overall accuracy 53.6%, user accuracy 100%). Irrespective of sensor spatial resolution, the reliability of detection varies with tree size. In northern Brazil, the probability of detecting the locations of extracted trees was 86% using TerraSAR-X Spotlight data [7]. However, 93% of larger trees (high biomass) were correctly detected compared to 76% of smaller trees (low biomass).

LiDAR is useful for detection of fine-scale (tree level) forest structure, and where repeat observations are possible, for detecting change. LiDAR depicts the 3D distribution of biological material in tree canopies and is highly sensitive to sub-canopy changes. Asner et al. [5] used LiDAR to analyse the variation in forest canopy gap distributions in the Peruvian Amazon. Relative density models (RDM), calculated as the relative percentage of LiDAR returns within a specified height stratum (1–5 m), revealed forest disturbance (e.g., trails and tree gaps) associated with selective logging in the western Brazilian Amazon [2].

#### Proxies

Proxy indicators, including logging roads, skid trails and log decks, can provide an estimate of the forest area considered (potentially) degraded, and can often be identified on moderate to high resolution optical and SAR imagery. Spectral fractions or spatial filtering of a monthly time-series of optical data can reveal the progression of logging roads, and combined to produce an annual synthesis of change. Proximity metrics such as distance to agriculture activity or infrastructure may be useful for delimiting (buffering) potentially degraded areas. Mapping outcomes may serve as a guide to identifying ‘hot spots’ for more detailed monitoring of changes in carbon stocks. Fragmented forest could also be a proxy for degradation [9, 17]. The area of intact forest, i.e., devoid of anthropogenic influence, can be identified using land cover maps generated from a consistent time-series of observations. A transition matrix between intact and non-intact forest and default or measured carbon stock change factors could then be used to estimate emissions and trends therein. Less frequent observations over long timescales are sufficient for degradation monitoring via proxies, however, the result may be of lower quality compared to direct mapping approaches [69].

Laporte et al. [52] used an extensive time-series of contrast-enhanced Landsat data to map the progression of logging roads in Central Africa. Roads were manually digitized and cross-checked by independent observers. The authors found the manual approach detected logging roads more consistently than semi-automated methods, particularly for older roads and when using images of lower quality. Forest degradation due to logging was delineated using a 1 km buffer around identified logging roads. Classification of IKONOS imagery (4 m) in the northern Republic of Congo revealed forest disturbance as related to logging skid trails and tree felling, which created large canopy gaps. Satellite-based monitoring provides the only practical and reliable means of monitoring legal and illegal logging activity in these remote areas.

Polarimetric and interferometric SAR (InSAR) can be used to identify roads and trails, even those hidden beneath the canopy. Solberg et al. [83] identified narrow forest roads as bands of low height values in TanDEM-X digital surface models (DSMs) in Norway. Williams et al. [95] observed tracks and roads through dense vegetation in Papua New Guinea using X- and P-band terrain corrected magnitude data overlaid on digital elevation models (DEMs) derived from GeoSAR data. Rauste et al. [74] devised an automated method of detecting new roads by linear feature extraction using the HV ratio and unsupervised classification of texture features extracted from ALOS PALSAR and TerraSAR-X data in the Republic of Congo. A user accuracy of 95% and overall accuracy of 70.4% was obtained using the L-band data, while values of 100 and 53.6% respectively were obtained using the X-band data. It was suggested that ALOS PALSAR data could be used routinely to map newly constructed roads as a proxy for forest degradation [74].

LiDAR is also used to identify logging roads and the potential area of disturbed forest. LiDAR derived RDMs revealed in increase of 17.1% in the area of roads, skid trails and landings, due to selective logging of tropical forests in the western Brazilian Amazon [2]. Disturbed areas were manually digitised by applying buffers to the centre lines of features identified in the 1–5 m RDM height stratum. The resulting 5 m resolution map was intersected with LiDAR-derived AGB (50 m) to identify the area of disturbed forest.

### Quantification of carbon stock changes

Quantitative estimates of forest carbon stock change are obtained by modelling AGB using remote sensing as input, or using a quantifiable proxy, such as a change in canopy height or tracking of forest successional stage.

#### Tracking of secondary forest dynamics

Characterisation of forest age and growth stage is one approach to tracking secondary forest dynamics. Together with knowledge of prior land use and disturbance history, this provides insight into current and future change in forest carbon stocks. A lengthy time-series is desirable, and most readily available from Landsat. Helmer et al. [29] applied the Threshold Age Mapping Algorithm (TAMA) to a discontinuous time-series of Landsat images (with an 11-date image sequence acquired between 1975 and 2003) to generate forest type age classification maps for secondary forests in Rondônia, Brazil. Forest age was mapped with an overall accuracy of 88% (Kappa 0.62). The algorithm is computationally efficient and self-calibrating, but requires testing in other forest types with different seasonality and disturbance histories [29].

The integration of SAR and optical data has also been investigated for the capacity to improve the discrimination of forest growth stages compared to using single or multi-date optical imagery alone. In Queensland, Australia, differentiation of forest regrowth stages was achieved by applying a threshold to image segments comprising ALOS PALSAR L-band HH and HV backscatter and Landsat-derived foliage projective cover (FPC; [59]). Mature (non-remnant) forest, early regrowth, intermediate and/or degraded forest and non-forest were mapped in the process, with an overall accuracy of 77.8% (Kappa 0.69). Confusion was greatest between the intermediate and mature stages. Rain events during image acquisitions affect the dynamic range of the SAR data, and images during the dry season are strongly preferred. Future monitoring within an operational framework is possible using data from ALOS-2 PALSAR-2 and Landsat-8. Historical vegetation dynamics can be also be assessed using archive Landsat FPC and L-band data from JERS-1 [57].

LiDAR also demonstrates potential for characterisation of forest successional stage and change in heavily modified forest by measuring changes in the vertical distribution of the woody components. The spatial extent of early, intermediate and late stage secondary dry tropical forest in Costa Rica was mapped using a 3-class ISODATA classification, and the change in vertical structure (including height) associated with each growth stage was assessed using the full waveform LiDAR data [11]. The accuracy of the LiDAR tree height estimates was assessed by comparing relative height (RH) metrics representing waveform energy quantiles (with RH100 the height above the ground of the highest reflecting surface; [11]) against field measured tree heights. The root mean square error (RMSE) of RH100 [11] was estimated at 1.34 m [coefficient of determination (r^2^) of 0.69, p < 0.001]. RH100 and RH75 were highly related to all successional stages (r^2^ of 0.79, 0.73 and 0.72 for late, intermediate and early stages respectively).

#### Canopy height change

While data from penetrative sensors, such as SAR and LiDAR, demonstrate a high sensitivity to forest structural parameters, including tree height, volume and AGB, their use in linking forest structural change to degradation in tropical forests is a relatively new application. Similarly, tree height estimation has been demonstrated using optical data and stereo- and photogrammetry techniques, but not used in monitoring programs to account for forest degradation. LiDAR is often used commercially in the forestry domain to evaluate the forest resource and reduce the field work load, but more recently, has found application in the R&D domain to study spatial patterns in the landscape and ecological processes. LiDAR estimates of canopy height are of a high accuracy (e.g., RMSE 1.34 m, [11]), and if repeat flights can be arranged, the technology is of immense benefit in capturing fine-scale forest dynamics at the tree level. Anderson et al. [2] used repeat LiDAR to measure structural change in selectively logged forests in the Western Brazilian Amazon. A simple differencing of two canopy height models (CHM) revealed the loss of 4.1% of tall canopy (>30 m) over the timeframe of image acquisition (~1.5 years). Key to the study was the use of certain LiDAR height metrics to quantify change associated with low-impact selective logging.

The ICESat GLAS was the only spaceborne LiDAR in operation between 2003 and 2009, and despite its primary function of monitoring changes in polar ice sheet elevations, GLAS data has provided regional estimates of forest height and AGB [53]. Estimates of Lorey’s height were obtained from GLAS data acquired over Gabon (r^2^ of 0.83, RMSE 3.3 m, n = 95; [65]). With no currently operational spaceborne LiDAR however, the method cannot be used for change detection. However, future capacity may be realised with the next generation LiDARs, including ICESat-2 and NASA’s Global Ecosystem Dynamics Investigation (GEDI).

Novel approaches are being developed that apply InSAR techniques to extract degraded forest areas from SAR data. A comparison of tree heights determined by inversion of a physical scattering model based on ALOS PALSAR correlation magnitude revealed disturbance in the forest as related to a change in vertical structure in Queensland, Australia [55]. DEM differencing using Cosmo-SkyMed Spotlight InSAR stereo data acquired over Cameroon and the Republic of Congo and the SRTM DEM revealed gaps in the canopy (i.e., loss of biomass) and roads as features of degradation [13]. Overall accuracies of 75–82% were obtained. InSAR DEM derived height and biomass change was demonstrated using TanDEM-X and SRTM DEM data acquired over Tanzania [84] and Uganda [85]. In Tanzania, the InSAR height changes correlated well with reforestation, degradation and deforestation events observed over the 11-year timeframe. In Uganda, height changes were mapped on a national scale, from which forest carbon stock changes and emissions were estimated [85]. Following removal of artefacts in the C-band DEM and correction of the X-/C-band penetration difference, the remaining bias was 0.9 mm and was variable upslope (8–16 mm). A comparison with Landsat derived Global Forest Cover data revealed a similar capacity for detection of forest carbon losses, but improved detection of carbon gains using the InSAR approach [85].

#### Above-ground biomass (AGB) change

Obtaining reliable estimates of AGB using EO data can be considered the holy grail of forest carbon science. Forest AGB is a quantifiable attribute, that when estimated continuously over time, and with reference to a baseline (reference level) representative of the mature (intact) forest state, could provide a useful indicator of degradation. The ability to characterise large-area biomass distributions would assist in providing national estimates of forest carbon stocks and GHG emissions.

Estimation of carbon stocks and change using EO data is technically challenging, largely because of the uncertainties associated with retrieval but also the prevailing environmental conditions, which often differ between two or more observation periods. Data acquired by penetrative SAR and LiDAR sensors show the most promise for quantifying AGB [3]. Precise, large-area estimates at the level of precision required for REDD+ carbon stock monitoring are yet to be achieved however. Changes in AGB can be quantified by (1) comparing two observations in time (*t*_1_ and *t*_2_) with coincident field data to model change in biomass directly, with differences at the pixel level corresponding to remote sensing observables at *t*_1_ and *t*_2_, and (2) modelling AGB for *t*_1_ and *t*_2_ separately and taking the difference.

Several studies have used LiDAR to discriminate degraded forest on the basis of AGB. LiDAR vertical profiles can be correlated with disturbance events or used to quantify the area subject to disturbance and the associated loss of carbon [67]. LiDAR profiles in Queensland, Australia, exhibited unique patterns when related to disturbance events (e.g., chaining, stem injection and logging) at discrete times over the Landsat record [58]. Full waveform measurements from overlapping scans captured by terrestrial laser scanner (TLS) allow the stand to be reconstructed in 3D, including woody debris (fallen logs and branches), and estimates of AGB obtained in situ [73].

**Fig. 3:**
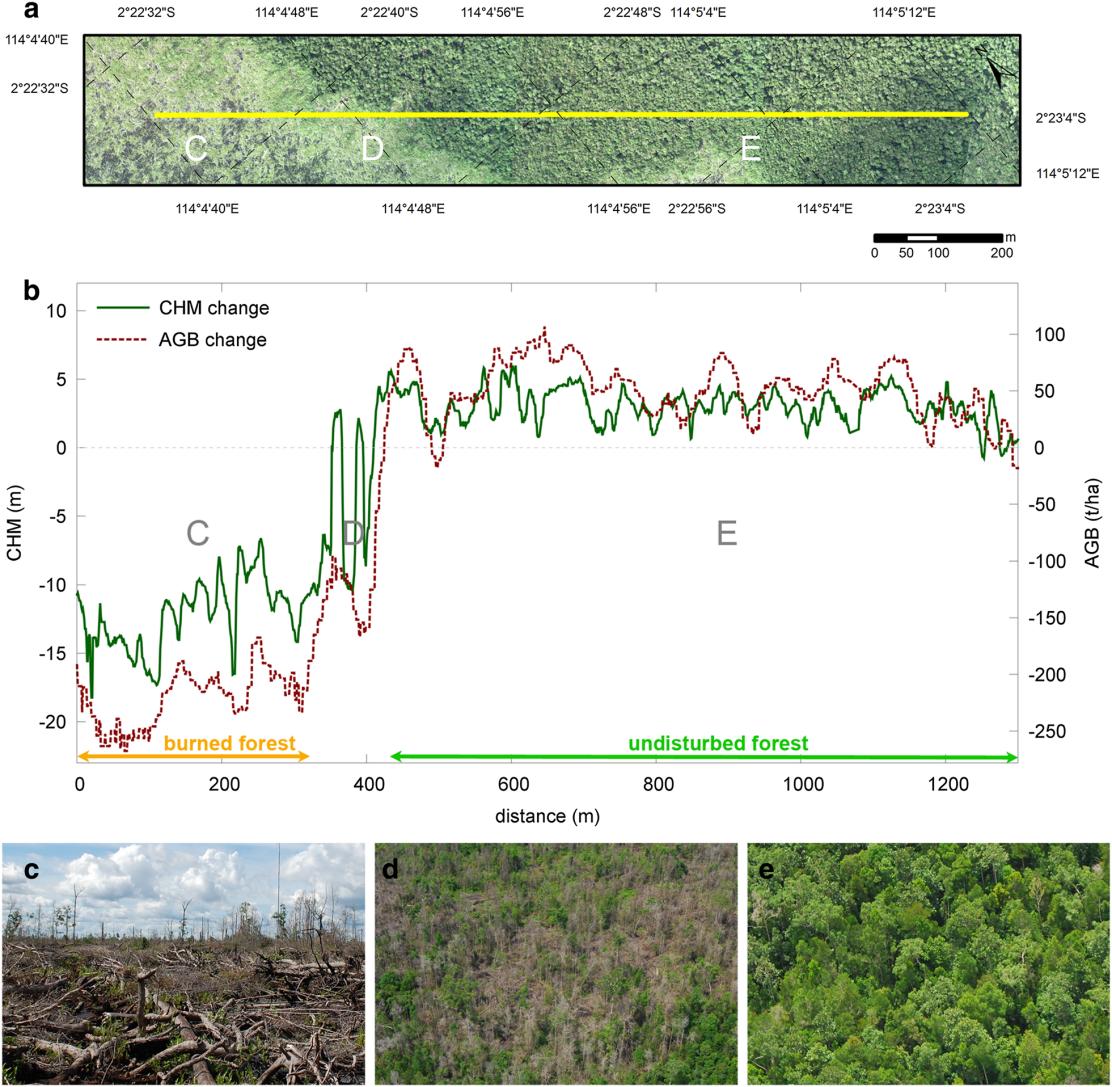
Use of multi-temporal LiDAR to quantify canopy height and AGB dynamics in tropical peatland forest: **a** Transect through burnt and adjacent undisturbed peat swamp forest. **b** Changes in canopy height and AGB associated with different forest conditions; and photographs of **c** burnt forest, **d** transition area and **e** undisturbed forest (Courtesy of [16])

Englhart et al. [16] used multi-temporal LiDAR acquired over tropical peatland forest in Kalimantan to quantify canopy height and AGB dynamics in unaffected, selectively logged and burned forests. AGB regression models had an r^2^ of 0.77 (Predictive Power of the Regression, PPR = 54.2 Mg ha^−1^) and 0.81 (PPR = 47.4 Mg ha^−1^) for the 2007 and 2011 LiDAR data respectively. Differences in AGB gain/loss and canopy height were evident between the forest conditions (Fig. 3). Selectively logged forest experienced an average loss of 55 Mg ha^−1^ within 30 m and 42 Mg ha^−1^ within 50 m of detected logging roads, while the mean canopy height increased by 0.5 and 1 m respectively [16]. Over the same 4-year timeframe, undisturbed forest saw, on average, a gain of 20 Mg ha^−1^ AGB and an increase of 2.3 m in canopy height, while burned forest lost 92% of its AGB. The potential of repeat LiDAR surveys for quantifying structural dynamics of relevance to REDD+ was clearly demonstrated.

Reliable estimation of the magnitude and extent of change from low intensity logging in the Brazilian Amazon using LIDAR was demonstrated by [2]. The multiple r^2^ was 0.7 and the standard error (SE) of the regression was 41.5 Mg ha^−1^. The change in AGB associated with the disturbed forest area was estimated at −17.9 ± 3.1 Mg ha^−1^ (p < 0.0001). Jubanski et al. [46] assessed the variability in AGB in lowland tropical forests in Kalimantan. LiDAR-derived height metrics correlated well with model-based estimates of AGB (r^2^ of 0.88, RMSE ± 13.79 Mg 0.13 ha^−1^). The point cloud exhibited unique signatures when related to disturbance events, e.g., illegal logging, and could be used to discriminate forest types, including peat swamps and disturbed forest. In a study located in boreal forest in south east Norway, a multinomial logistic regression model was used to predict the change class (e.g., deforestation, degradation, no change) from repeat LiDAR data acquired over an 11-year period [67]. The change categories were then used as post-strata in estimating the net change in biomass. Estimates of AGB loss in the degradation post-stratum were estimated with an SE ranging from 5 to 8.4 Mg ha^−1^ (r^2^ of 0.88–0.98). The study demonstrated the potential of LiDAR to distinguish between activity-based change categories that are highly relevant to international reporting including REDD+.

Arguably the main observational gap, in terms of available EO technology, in retrieving forest AGB is a spaceborne LiDAR. Airborne LiDAR is currently not sufficiently affordable to governments to acquire multi-year and wall-to-wall, other than for local REDD+ projects. LiDAR-assisted approaches have, however, demonstrated the capacity to obtain almost the same level of precision through the integration of a 1–5% LiDAR sample with wall-to-wall satellite data, and so facilitate national estimates of AGB [20]. The integration of ICESat GLAS with optical and/or SAR data has been successful in estimating AGB in a range of forest types [60, 65, 71]. Quiñones et al. [71] produced a map of AGB for Kalimantan using ALOS PALSAR and GLAS derived canopy height. A 17-class vegetation structural type (VST) map was first produced using ALOS PALSAR dual polarisation imagery. ICESat heights were then extracted for each of the VST classes and histogram matching applied to integrate the SAR and LiDAR measurements. Allometric equations were used to convert the histograms to AGB estimates. A comparison with field based estimates of biomass revealed an SE of 53.2 Mg ha^−1^ (n = 52) in the 50 m resolution map for AGB ranging up to 520 Mg ha^−1^. The rate of AGB accumulation in secondary forests in Rondônia, Brazil, was estimated by combining Landsat derived forest age with GLAS derived estimates of AGB [29]. A significant relationship was observed between secondary forest age and AGB (r^2^ of 0.6, p < 0.0001, n = 26). There was good agreement between the satellite-derived average biomass accumulation rate of 8.4 Mg ha^−1^ year^−1^ and ground-based measurements for young secondary forests. With future spaceborne LiDAR, these methods will be available for operational monitoring of biomass change in all forest types.

The sensitivity of SAR to canopy structure and biomass can be exploited to map changes associated with young and degraded (low biomass) forests. SAR sensitivity to biomass varies with frequency, with C- and X-band tending to saturate at low biomass levels (25–50 Mg ha^−1^), L-band at around 50–150 Mg ha^−1^ and P-band at 100–200 Mg ha^−1^ [8]. Data from, for example, ENVISAT ASAR and ALOS PALSAR have been used to retrieve AGB in young and degraded forests, but are less useful for mature, higher biomass forests [8, 63]. Ryan et al. [78] combined time-series ALOS PALSAR data to generate carbon stock change maps for Mozambique. Changes in carbon densities as little as 12 MgC ha^−1^ over 3 years were detected with 95% confidence, allowing characterisation of carbon stock loss from deforestation and degradation at a new level of detail. Data fusion approaches may help overcome sensor specific limitations such as saturation, operating modes and temporal gaps [10].

Fully polarimetric and InSAR data affords greater detail on forest structure, and may provide further insight into degraded forests. In Tanzania, AGB change was predicted based on InSAR height change from TanDEM-X and SRTM DEMs with an accuracy of 67.2 Mg ha^−1^ (51%; [84]). Solberg et al. [85] demonstrated wall-to-wall forest carbon change mapping in Uganda using TanDEM-X (2012) and SRTM (2000) DEM data. Canopy height decreased by 2.6 cm year^−1^, corresponding to an annual CO_2_ emission of 20.7 Mg ha^−1^ (±8.5 Mg ha^−1^ at the 95% confidence interval). Temporal measurement of InSAR height and volume, as linked to AGB, may provide countries with a practical approach to estimating forest carbon stocks and emissions arising from gradual processes such as degradation and regrowth [85].

## Operational readiness of EO sensors for monitoring degradation

The EO data requirements for monitoring forest degradation will vary depending on the type of degradation or proxy, and will be greater the more activities are to be monitored under the REDD+ spectrum. Resolving change on a small Minimum Mapping Unit (MMU) will necessitate the use of high to VHR satellite data, the cost of which may be limiting to countries wanting to implement an operational wall-to-wall monitoring system. The use of coarser resolution data may reduce the data demand, but certain areas of activity may escape detection. Multi-sensor data are required to monitor a broad range of degradation types, with sensor capability targeted at both the detection of canopy cover change (optical/SAR) and sub-canopy structural change (SAR/LiDAR).

The operational readiness of the technology, in terms of satellite data availability, robustness of methods, large-area demonstrations and country operational examples, is evaluated in Table 2. Forest degradation mapping methods are largely considered in an R&D phase [21], with large-scale demonstrations (i.e., sub-national to national level), scaling from project to national level, automation of methods and tuning of algorithms for different forest types needed to pre-/operationalise methods for use in a REDD+ monitoring context. The lack of systematic observations by key EO sensors has hampered methods development; as such, large-scale demonstrations are few. Numerous case studies have, however, demonstrated a high potential for retrieving activity data on forest degradation, as well as uncovering history of land use and other causes of disturbance using EO data. Data fusion can assist in mapping degradation, but obtaining near-coincident data is difficult with little to no coordination of SAR and optical satellite observations by space agencies. Countries need access to low cost, high to VHR data to detect changes in forest cover and carbon stocks and so include estimates of emissions from degradation in their forest inventorys. Access to free high resolution optical data has only recently become available with the launch of Sentinel-2. Other high resolution data, including SAR, are only available from commercial suppliers. With the exception of Sentinel-2, high resolution data are tasked on request, often resulting in fragmented spatial and temporal coverage. There may be a case for using a sample of high to VHR images within a wall-to-wall monitoring system.

**Table 2:** Operational readiness of current EO sensors for monitoring forest degradation

Degradation type	Resolution	Data source	Mode (optimal)	Sensor (Launch date)	Geographical data coverage	Methods developed and tested	Large area demonstrations	Country operational examples
Broad-scale degradation (fire/logging/regrowth/disease)	Moderate (10–100 m)	Optical	VNIR-SWIR	Landsat (1972-)	Yes	Y	Y	Y
				Landsat-8 (2013-) CBERS-4 (2014-)				
		L-band	DP (10 m)	ALOS-2 PALSAR (2014-)	Global 2 obs year^−1^; Tropical 4 obs year^−1^	Y	Y	N
			DP (50 m)		Tropical 9 obs year^−1^			
		C-band	SP (20 m) DP (20 m)	Sentinel-1A/1B (2014-/2016-)	Global, monthly or better (^a^S1 BOS)	Y	N	N
			DP	RADARSAT-2 (2007-)	Requests required			
	High (5–10 m)	Optical	VNIR-SWIR	Sentinel-2 (2015-) SPOT-6/7 (2012-/2014-) RapidEye (2009-)	(^a^S2 BOS) Requests required	Y	N	N
		X-band	SM 3D TDM	TerraSAR-X (2007-) TanDEM-X (2011-)	Requests required	Y	N	N
Fine-scale degradation (selective logging, roads, encroachment)	VHR (<5 m)	Optical	VNIR-SWIR	GeoEye (2008-) WorldView-3 (2014-)	Global (heterogeneous)	Y	N	N
		X-band	SM/SL SM	TerraSAR-X (2007-) COSMO-SkyMed (2007-)	Requests required	Y	N	N
		X-band	3D TDM	TanDEM-X (2011-)	Global (2 times)	Y	N	N
		LiDAR (airborne)	Full waveform	N/A	No	Y	Y	N

^a^Baseline observation scenario (BOS) for Sentinel-2: systematic observation over Europe, Africa and Greenland; other land surfaces every 20 days; BOS for Sentinel-1 IW: Forestry and Agriculture Priority areas, every 12–24 days

Optical imagery is widely used and offers the most operational capability today. Open access to the USGS Landsat archive has spurred methods development using moderate resolution data. The long temporal archives are important for baseline generation (i.e., establishing forest and emissions reference levels) and investigating land use history [18]. Data from previous generation L-band SAR systems (e.g., JERS-1 from mid 1990s, and ALOS PALSAR between 2007 and 2011) can also be integrated to observe historic and more recent change. Brazil’s forest degradation monitoring system is reliant on free access to Landsat and CBERS data.

Higher resolution optical data is available through commercial missions (e.g., SPOT-6/7, RapidEye, GeoEye). The high cost can be prohibitive, but the high resolution is a requirement for detection of fine-scale degradation processes. High temporal frequency is also a requirement for capturing discrete events or rapid change (regrowth) in dynamic landscapes. Ideally, one cloud-free seasonal coverage is required for tracking forest disturbance. We should see a rise in methods development using Sentinel-2 data in the near future. The high temporal frequency, now, and with the constellation (5 days; Sentinel-2B launched successfully on 7 March, 2017), will boost capability in severely cloud-affected tropical regions. Inclusion of red edge bands and the short wave infrared (SWIR) will support the retrieval of information on forest condition and so support forest degradation monitoring efforts. Free and open access to data will greatly assist the establishment of long-term monitoring programs.

Of the radar missions, Sentinel-1, ALOS-2, RADARSAT-2, TerraSAR-X/TanDEM-X and COSMO-SkyMed are currently in operations. Both ALOS-2 and the Sentinel-1A and 1B satellites operate under a pre-defined observation plan, and provide global systematic observations over land areas, whereas recommendations to X-band SAR data providers are required to support future acquisitions of high resolution SAR data. JAXA’s Basic Observation Scenario (BOS) for ALOS-2 comprises amongst others, 2 dual polarisation (DP) observations at 10 m per year over global land areas, and 4 (DP) and 9 ScanSAR (50 m) observations per year in tropical regions for forest and wetlands monitoring [76]. Estimates of forest height and biomass (up to ~150 Mg ha^−1^) are possible using L-band data, but the level of precision is unlikely to meet the requirements of REDD+. L-band capability for retrieving AGB may be extended with polarimetric interferometry (PolInSAR) or its integration with other EO data. P-band SAR offers greater penetration depth, but there are no currently operational P-band SARs. The European Space Agency (ESA) P-band BIOMASS mission is scheduled for launch around 2021 and will provide support for global carbon budgets. While the cost of ALOS-2 data has been found to limit operational use of L-band SAR data [75], alternative sources of SAR data will be realised with the (2017/2018) launches of the Argentinian SAOCOM-1 constellation, with dual-season pan-tropical DP and full polarimetric observations at L-band [77], and (around 2021) by the US-Indian NISAR mission, which is planned to comprise global DP observations with very high temporal repetition [45].

The bulk of Sentinel-1A and 1B terrestrial observations are undertaken in interferometric wide-swath (IWS) mode, and provide 20 m resolution single (SP) or DP data [79]. RADARSAT-2 data are commercial and acquired on demand, although broad area DP coverage every 26 days is available over certain regions. Only few studies have investigated the utility of C-band observations for forest degradation monitoring. Typically, DP and a high frequency of observations is required to improve estimates of forest cover change, particularly in regrowth areas where canopy closure is rapid. The 6-day repeat cycle with the two-satellite Sentinel-1 constellation has the potential to provide this capacity. High resolution SAR data is available commercially at X-band. Improved access to X-band SAR observations from TerraSAR-X, TanDEM-X and COSMO-SkyMed would stimulate research in estimating structural parameters and tracking disturbance. The very high spatial resolution is well suited to detecting tree level change, including canopy gaps in selectively logged forests, and roads and other proxy measures of degradation.

Airborne LiDAR is a promising technology at the project scale, but not yet considered operational for tropical forest monitoring [1]. Airborne LiDAR is available on a commercial basis, and together with TLS, can be deployed when needed. Published studies, in and beyond the tropics, show that there is some experience in monitoring degradation, with repeat LiDAR surveys used for spatially explicit modelling of forest carbon stocks and change. Unlike SAR sensors, there is no saturation at high levels of biomass [30]. The next generation LiDARs, including NASA’s Global Ecosystem Dynamics Investigation (GEDI), could revolutionize global estimates of forest structural parameters, including forest height and AGB, and so contribute to REDD+ monitoring and evaluation [60].

To summarise, operational forest degradation monitoring is hampered by the lack of continuous and coordinated multi-sensor satellite coverage, high cost of VHR data and lack of national-scale methods [14]. Improved access to affordable dense time-series optical and SAR data over tropical forests is essential to support research efforts to extend methods to pre-operational and operational use, and to maintain existing national forest monitoring systems. This requires a commitment by space agencies for systematic and coordinated observation of forested areas on a sustainable basis and with an open data policy. Satellite permanence entails both government and private sector support and recognition of the important role of satellite-based monitoring of global forest and land cover. Access to calibrated, orthorectified (‘analysis ready’) satellite data would increase uptake and use by non-experts. Ensuring access to archive data would extend the time-series available for establishing forest reference levels and understanding trends, and when integrated with more recent data, enable prediction of future impacts and guide restoration efforts. Countries will need to prioritise their monitoring needs, depending on the significance and drivers of degradation, and their available resources. As new data, methods and capabilities emerge, ideally a system of continuous improvement is in place in mandated country organizations to improve REDD+ monitoring efforts.

## R&D and capacity building needs: the way forward

In the context of REDD+, there still exists a need for the design of an operational framework that includes EO-based methods of detecting forest disturbance and quantifying change in carbon stocks over long time-scales and with documented uncertainties and at national scale. The trade-offs that exist between monitoring costs and precision, and how this translates to REDD+ benefits, need to be better documented. Whether targeted hot spot analysis can improve the efficiency of monitoring efforts for estimating emissions should be investigated [32].

While there are well-established time-series methods for tracking forest disturbance, these have largely been developed using moderate resolution EO data. Transference of methods to higher resolution data requires testing and in a range of different forest types and AGB strata. The archive of high resolution, high frequency (bi-/monthly) observations is increasing (e.g., with Sentinel-1/2), and will provide useful data for case studies that improve our understanding of the factors affecting the purity of the degradation signal, the effects of seasonality on vegetation response and the ability to separate real change from inter-annual variability [51]. The majority of forest degradation mapping methods available now are also focussed on single-sensor solutions. Additional research effort is needed on SAR-optical fusion for improved detection and mapping of degraded forests [94]. The results of R&D should be included in technical guidance with specific reference to the upper and lower limits (e.g., change in percent canopy cover or tree size, [37]) within which degradation can be detected.

Broad-scale remote sensing approaches to the retrieval of AGB and over long time-scales are not yet considered operational at national scale. The application of any one method in a monitoring context for forest degradation is yet to be established. Further R&D should focus on the use of archive data for determining baseline carbon stocks [57], and tracking of secondary forest dynamics using SAR-optical data fusion in a range of forest types and with varied disturbance history [29, 59]. Mapping approaches that link changes in canopy height with degradation warrant further investigation and repeat monitoring studies will be possible with future launches of GEDI and ICESat-2. InSAR DEM differencing techniques are still in their infancy and require further testing over larger areas and for a range of degraded forests [13, 84, 85]. For those methods that attempt to quantify changes in AGB, it is not yet known whether modelling the change in AGB directly or differencing two modelled estimates is the optimal approach. Likely this will be sensor specific, and so a better understanding of the imaging parameters (timing and frequency of measurement) and the level of precision achieved is needed. Promising approaches have been identified that may lead to improvements in AGB change estimates. The reliability and transferability of LiDAR-based retrieval algorithms (using next generation LiDARs), SAR-based methods [84, 85] and data fusion (SAR-Optical, SAR-Optical-LiDAR, [10]) approaches should be investigated in the context of estimating change in degraded and regenerating forests. Further investment in LiDAR-assisted estimation of AGB [20], including improvements to sampling design and estimators, may provide a method suitable for regional estimation of forest height and biomass, which could form part of a degradation monitoring system [20]. Lastly, additional R&D effort is required on improvements to integrated EO/in situ approaches, including uncertainties in upscaling point, transect and area data [46]), stratification schemes for improved precision of AGB change estimates and maximising the use of sample data (e.g., when there are limited or missing samples).

A more coordinated research effort is paramount to developing and demonstrating robust and consistent approaches to forest degradation monitoring in support of NFMS and REDD+. Global initiatives are underway to support R&D in high priority topics such as forest degradation [e.g., 23]. Once methods reach maturity (i.e., are considered in a pre-operational or operational phase of development), are well calibrated and validated and applicable in the sub/tropical forest context, it is anticipated they will be implemented in operational NFMS. This is highly dependent on available resources, and will vary by country. Just because the technology is available, this does not automatically translate into its operational use [6]. The ideal scenario comprises (1) a commitment from space agencies to systematic acquisition of appropriate and free or low-cost satellite data over all forested areas, (2) the outcomes of R&D to be integrated into training materials and capacity building initiatives, (3) donor support and understanding of the science behind the reporting and what is realistic and achievable, and (4) Government support for sustainable MRV programs and national forest inventory.

## Concluding summary

Although not exhaustive, this review has captured a range of practical approaches, and identified some of the limitations associated with the remote sensing detection and monitoring of forest degradation. The extensive Landsat archive and available time-series methods provide a good opportunity to understand land use history and establish forest reference and emissions levels, against which to monitor change and determine the relevant time-scales over which degradation occurs. Canopy cover change can be monitored using a dense time-series (comprising optical or SAR observations or a combination of both), while spectral fractions, unmixing or classification can be used to separate degraded and intact forest. Changes at tree level (e.g., canopy gaps) can be detected using high to VHR data. Proxies, including logging roads and log decks have also proven useful in identifying the area undergoing change. Timely and routine detection of degradation requires frequent observations of appropriate satellite data. Open data policies will support both routine monitoring efforts and those that identify degradation before it turns to deforestation; the latter which could be built into an early warning system. Estimation of AGB is difficult at national scale and current methods using satellite data do not meet the level of precision required for REDD+ reporting. An interim solution might involve LiDAR-assisted sampling or using L-band SAR data to gain an overview of biomass strata in low biomass and degraded forests. Novel techniques are being developed using InSAR and LiDAR data and looking at change in vertical structure and volume. Future spaceborne LiDAR will improve the capacity to monitor the extent and magnitude of AGB change in degraded or disturbed forests.

Degradation can be a gradual process and a firm understanding of the drivers and impacts of change is needed to devise the optimal monitoring strategy, guide policy development and identify areas for possible restoration [32, 93]. Addressing the afore-mentioned R&D and capacity needs will allow for full-scale implementation of an MRV system, and so allow those countries who wish to fully implement REDD+ to move towards improved forest management and reduced emissions.

## Abbreviations

MRV: measurement, reporting and verification
UN REDD+: United Nations Reducing Emissions from Deforestation and Forest Degradation
IPCC: Intergovernmental Panel on Climate Change
SAR: synthetic aperture radar
LiDAR: Light Detection and Ranging
AGB: above ground biomass
GHG: greenhouse gas emissions
CO_2_: carbon dioxide
UNFCCC: United Nations Framework Convention on Climate Change
EO: Earth Observation
R&D: research and development
BFAST: Break detection For Additive Seasonal Trends
NDVI: Normalized Difference Vegetation Index
LandTrendr: Landsat-based Detection of Trends in Disturbance and Recovery
NBR: Normalized Burn Ratio
CCDC: Continuous Change Detection and Classification
DI: Disturbance Index
SMA: Spectral Mixture Analysis
INPE: National Institute for Space Research
VHR: very high resolution
FAR: false alarm rate
RDM: relative density model
InSAR: Interferometric SAR
TAMA: Threshold Age Mapping Algorithm
FPC: foliage projective cover
RMSE: root mean square error
CHM: canopy height model
DSMs: digital surface models
DEMs: digital elevation models
TLS: terrestrial laser scanners
SE: standard error
MMU: Minimum Mapping Unit
SWIR: short wave infrared
BOS: Basic Observation Scenario
DP: dual polarisation
PolInSAR: polarimetric interferometry
ESA: European Space Agency
IWS: interferometric wide-swath
SP: single polarisation
NFMS: national forest monitoring systems
